# Sports medicine service and popular sports: a multidisciplinary contribution to a specific component of the health system of the German Democratic Republic

**DOI:** 10.3389/fspor.2026.1619573

**Published:** 2026-03-12

**Authors:** Fabian Krug, Fabian Standl, Heribert Stich

**Affiliations:** 1 Pettenkofer School of Public Health, University of Munich-LMU, Munich, Germany; 2 Department of Public Health Medicine, Augsburg, Germany; 3 Institute for Medical Informatics, Biometry and Epidemiology (IMIBE), University Hospital Essen, Essen, Germany; 4 Technical University of Munich, TUM School of Medicine and Health, Graduate Center of Medicine and Health, Munich, Germany; 5 Institute for Medical Processing, Biometry, and Epidemiology-IBE, University of Munich-LMU, Munich, Germany; 6 Department of Public Health Medicine, Landshut, Germany

**Keywords:** German Democratic Republic, health care system, health promotion, health services research, popular sports, sports medicine service

## Abstract

Against the background of decreasing levels of physical activity in industrialized nations, the promotion of sports as a preventive medical concept is of current importance. The so-called Sports Medicine Service (“Sportmedizinischer Dienst”; SMD) in the former German Democratic Republic (GDR) pursued this objective with varying degrees of intensity. Through a systematic literature review on a conventional and internet-based basis, original documents and scientific articles were made available. In 1963, a significant milestone was reached with the establishment of a specialist in sports medicine and the development of the SMD as a care institution. The initial focus was on mass sport as a public health preventive measure. Through defined tasks, a centralized organizational structure, general medical sports advice and district centers for medical sport consultation, as many East Germans as possible were to receive support in their sporting activities. However, from the 1970s onwards, for ideological reasons, national professional sport was increasingly prioritized within the SMD, which in some cases led to a substantial reduction in resources for amateur sport. With the reunification of the two German states, the SMD was swiftly dissolved in 1990 without replacement. From a neutral but critical perspective, the dissolution of the SMD without replacing the health-promoting aspects of amateur sport and without technical reflection was inappropriate. A well-thought-out use of health-promoting elements of the SMD within a democratic society would be an approach to remedy this deficit, which calls for renewed initiatives to promote amateur sports and subject-related research projects.

## 1 Introduction

Over the past few decades, the importance of health-promoting and preventive measures has been repeatedly discussed by a wide variety of actors in the field of public health (1). Especially with regard to health science topics such as lack of exercise (2) by people in industrialized nations, there is a clear need for both research and action.

Against the backdrop of the alarming developments in physical inactivity, it is worthwhile to look back at the “Sports Medicine Service” (“Sportmedizinischer Dienst”; SMD) in the former GDR. The SMD was a state-run, nationwide system of medical sports care for the population with sporting ambitions (3–5). Nationwide, qualified contact persons were available for every recreational athlete. In most cases, many of these experts initially worked on a voluntary, unpaid basis, and eventually transitioned or a paid part-time basis in a so-called Z (supplementary) relationship. From 1970, the district sports physicians were (6, 7) assigned further areas of responsibility, such as the care of young athletes admitted to the training centers, and extended their sports medicine activities to other key areas within the SMD (8, 9), while still maintaining a high scientific level in prevention, rehabilitation, recreational and performance medicine by international standards (10, 11).

The aim of this study was to provide an overview of the SMD with a focus on recreational sports and to evaluate what approaches popular sport can offer as a population-based preventive measure in democratic industrialized nations.

## 2 Methods

The methodological approach to literature research was structured according to the principles of a scoping review (12). Although not all formal requirements were implemented, the multi-stage process aimed to systematically, transparently, and comprehensibly narrow down the relevant body of literature. The combination of quantitative screening steps and content relevance assessment enabled a structured yet theory-driven selection of suitable primary sources and contextual literature. A systematic literature review on a conventional and digital basis (13) was carried out by three independent persons to address the topic. At the beginning of the conventional literature search, the search terms “Sportmedizinischer Dienst”, “DDR”, “Sportmedizinischer Dienst”, “Facharzt*in für Sportmedizin”, “Gesundheitsförderung”, “Prävention”, “Prophylaxe” and “Breitensport” were used to identify useful sources using the Google search engine. In addition, the search term “Sportmedizinischer Dienst” was used to search for topic-related literature in the “Online Public Access Catalogue” (OPAC) of the university libraries (UB) of the Ludwig-Maximilians-University Munich (LMU) and the University of Augsburg. Only six results were found in the LMU University Library and 35 in the Augsburg University Library. In addition, digital searches (13) with selected search terms like used in conventional literature search were conducted in the databases LIVIVO (98 results), Google Scholar (218 results), Pubmed (78 results), Epistemonikos (39 results) and Embase (55 results). This approach made it possible to identify both original documents and secondary literature on the various stages in the development of the SMD, and it became apparent that secondary literature was rare. In addition, a review of so-called grey literature was carried out in Google Scholar (14). The presentation of results is based on a systematic qualitative content analysis of a total of 32 documents, which were identified in the scoping review and selected according to their relevance to the topic. The analysis is based on the methodological approach described by Kuckartz (15). A formal bibliometric analysis was not conducted, as the available literature was heterogeneous in publication type, time period, and indexing status, and many relevant sources consisted of archival or grey literature that is not amenable to standard bibliometric approaches.

## 3 Results

### 3.1 The starting conditions for the establishment of the SMD from 1949 to 1963

The “Law on the Participation of Youth in the Development of the German Democratic Republic and the Promotion of Youth at School and at Work, in Sport and Recreation” (“Gesetz über die Teilnahme der Jugend am Aufbau der Deutschen Demokratischen Republik und die Förderung der Jugend in Schule und Beruf, bei Sport und Erholung”) of February 8, 1950 underpinned the importance of sport, physical activity and recreation for young people in East Germany, as only “…an educated, physically healthy, strong youth, progressive in its views and aspirations, will secure a united, democratic and peace-loving Germany” (16). This obliged all organs of the state administration “…to promote the further development of the democratic sports movement and hiking in the GDR as well as the education of a physically and mentally healthy young generation and to give them great opportunities for enjoyment and recreation” (16). As a result, the promotion of sporting activities was seen as a state task and purpose, focusing on the physical and mental health and well-being of young people. For example, the sports performance badge “Ready for work and for the defense of peace in the German Democratic Republic” (“Bereit zur Arbeit und zur Verteidigung des Friedens der Deutschen Demokratischen Republik”) was created in 1950, which was intended to serve as an incentive for the development of a physical culture in the GDR (17). Furthermore, § 39 of this law envisaged the construction of a “University of Physical Culture” (“Hochschule für Körperkultur”) to train lecturers for the institutes of physical education, sports teachers and coaches with a capacity of 400 students (16). Through such subsidies, the state exerted a strong influence on the health behavior (behavioral prevention) and living conditions (structural prevention) of the East German population.

### 3.2 Order for the organization and implementation of medical sports care

On 20 November 1953, the Ministry of Health issued the “Order on the organization and implementation of sports medicine service” (“Anordnung über die Organisation und Durchführung der sportärztlichen Betreuung”) (18), which was a logical consequence of the demand for sports medical advice and care for the population as a state objective (18, 19). This marked the beginning of the unique sports medicine service system in the GDR (18), even though the final implementation of the SMD took almost ten years. Table 1 provides an overview of the range of tasks performed by sports physicians before and after the SMD was established.

**Table 1:** Tasks of the sports physician before and after the introduction of the SMD (3, 18).

Tasks before the introduction of the SMD	Additional tasks *after* the introduction of the SMD
Regional responsibility for sports medical care	Central, state organizational structure Sports medicine service
Advice to the sporting population;	Realization of a General practitioner system;
Realization of the legal provisions on exemption from school sports;	Regular observation in training;
Carrying out fitness for sport examinations;	Contact person for athletes;
Safeguarding sports events;	Training design;
	Knowledge and skills in relation to sport-appropriate general and specialized health care in the practice of physical culture and sport;
	Knowledge and skills in the assessment of performance diagnostic examinations to record and evaluate physical performance;
	Knowledge and experience of active co-operation in increasing physical performance and exercise tolerance;
	Knowledge of the use of the means and methods of physical culture and sport to maintain and consolidate the health and develop the performance of all citizens;

### 3.3 SMD range of tasks

The introduction of the medical specialist for sports medicine (4, 20) resulted in fundamental changes for the work of doctors practicing sports medicine and was oriented towards the objectives of the SMD, which was laid down in the SMD statutes of 10 Sptember 1963: “The Sports Medicine Service is a medical institution for the sports medical care and control of the sporting population and contributes to physical culture and sports as a means of improving health and performance and as part of a healthy lifestyle becoming increasingly effective” (21). This interpretation is worth mentioning from a health science perspective, as the focus is directed towards improving even more on improving health and performance as well as a healthy lifestyle, for which the areas of “physical culture” and “sport” were identified as important instruments.

The main responsibilities for the SMD included the organization of sports medical care and monitoring of the sporting population within the associations of the “German Gymnastics and Sports Federation” (“Verbände des Deutschen Turn- und Sportbundes”; DTSB) (first level). In addition, the methodical guidance of the organs and institutions the oversight of the Ministry of National Education and the State Secretariat for Higher and Technical Education in sports medicine, as well a the supervision of students in collaboration with health system entities such as school and youth doctors, was determined (21). The central objective was to involve ever broader sections of the population, but especially all children and young people, in regular sporting activity and a healthy lifestyle (21). The SMD’s main areas of responsibility therefore the organization, sports medical care and monitoring the sporting population. The statutes also provided information on the sub-areas into which these tasks were to be divided. One of these sub-areas was general basic sports medical care (organization of medical care for sporting events and sports medical care, which mainly involved carrying out fitness for sport examinations, school sports exemptions, ongoing health checks and prophylactic, therapeutic and sports hygiene measures). The so-called second and third organizational levels of the SMD related to the special sports medical care for competitive and elite athletes of the DTSB sports associations and the highly specialized sports medical care for complex performance diagnostics in elite sports and for competitive athletes who were injured or ill (21). In order to accomplish these tasks, the SMD was required to work closely with the organs of the state apparatus and social organizations, but above all with the state organs of the health system, the executive boards of the GDR Gymnastics and Sports Federation and the German Society for Sports Medicine of the GDR (21).

### 3.4 Organizational structure of the SMD

**Figure 1:**
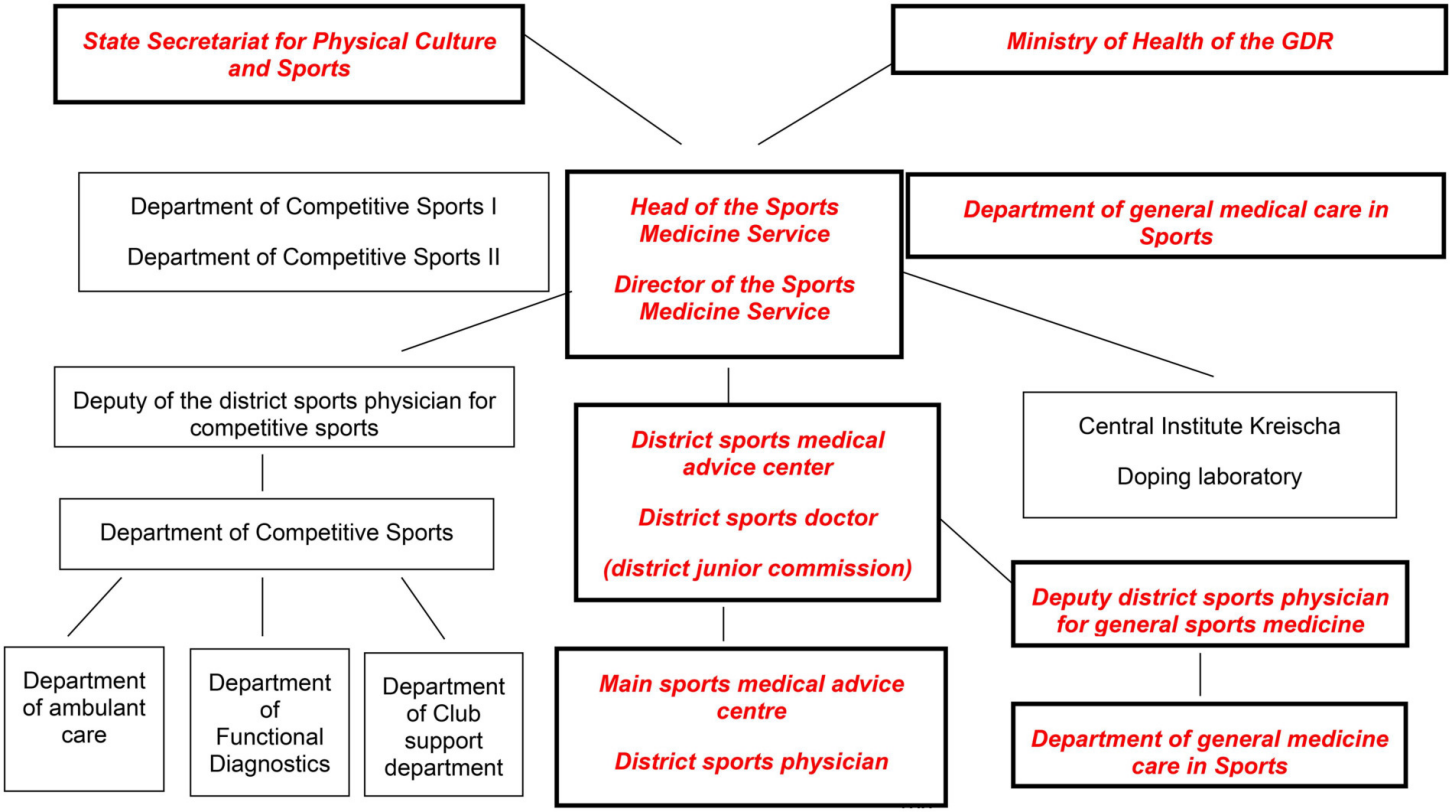
Structure of the sports medicine service (SMD) [modified after (22), p. 214]. SMD facilities that prioritize popular sports are highlighted in bold italics and in red colour.

The organizational structure of the SMD can be seen in the figure. The SMD was headed by a Director of the SMD, who was subordinate to the Ministry of Health of the GDR and the State Secretariat for Physical Culture and Sport (Figure 1).

### 3.5 General sports medicine service

This area mainly served to cover leisure and recreational sports (popular sports), but was always linked to the area of competitive sports (10). The district sports medical advice centers had a significant influence on the work in general sports medical care (22, 23) with the sports medicine specialists working there (4, 20). In order to meet the ever-increasing challenges following the introduction of the SMD in the area of general sports medical care, the district sports medical advice centers—as well as all other SMD departments—had to be set up more professionally. This included increasing the number of jobs in sports medicine and the number of staff and equipment in the districts. In order to analyze the prevailing structures and processes and to understand the role of the SMD in this scenario, it is worth taking another look at a draft law of the GDR. The agreement on further sports medical care for the sporting population from November 1979 made it clear that initially every GDR citizen should be given the opportunity to take advantage of the free sports medical care and advice necessary for their desired sporting activity (23). The SMD was responsible for organizing the sports medical care and providing content-related guidance to the state healthcare facilities involved in the respective districts (23). The specific tasks of the SMD thus included the sports medical care of all athletes training in sports clubs, children's and youth sports schools, training centers and training bases. Accordingly, all GDR citizens who practiced physical culture and sport in the area of leisure and recreational sports (“mass sports”) to promote performance and health (in the family, at work, individually, in social institutions or in centers for active recreation and health sports) were able to obtain advice from the health institution responsible for them (e.g., district sports medical advice center) on the most appropriate form and design of health training from their responsible health institution personally. It's worth noting the regular health checks of citizens in the district advice centers, where sports medical advice could also be obtained (23).

In summary, the central areas of general sports medical care were

- the organization and guidance of the district sports medical advice centers in questions of sports medical care for young athletes,
- the guidance of the association doctors of the non-Olympic sports associations,
- providing guidance and further development on issues relating to school sports and school sports exemptions
- the development of basic sports medicine materials,
- the organization of medical and hygienic care at major events (GDR gymnastics and sports festivals, GDR children's and youth spartakiads, district children's and youth spartakiads).

### 3.6 District sports medical advice center

The district sports medical advice centers were responsible for fulfilling the tasks of the SMD and were staffed full-time from 1968 by a district sports physician and one or two medical assistants (3). The work in the district advice centers was usually carried out by the district sports physician in collaboration with a nurse. There was usually also a part-time cleaner. The nurses were responsible for carrying out exercise and resting ECGs, ergometry, muscle function tests and anthropometric measurements. The district sports physician (head of the district sports medical advice center), on the other hand, was responsible for:

- the planning, management and implementation of sports medicine work in the district,
- organizing and guiding the content of sports medicine consultations in close cooperation with the public health facilities in its territory
- sports medical checks on competitive athletes and sportspeople who required special sports medical care due to their sporting activities or health conditions.

### 3.7 Popular sport in the GDR—an exemplary selection

In addition to so-called popular sport, competitive sport became increasingly important within the political system and the health system of the GDR, which led to a reduction in resources being made available for popular sport. However, this particular problem will not be discussed further here, but the focus will be on “mass sport”.

At the beginning of the SMD, the term popular sport was referred to as “mass and popular sport”, which gave way to the term “leisure and recreational sport” (“Freizeit- und Erholungssport”) in 1968 (24). The ideological objectives were the general function of sport as part of the unified process for the construction and development of socialism (25, 26). As a result, these sporting activities were intended to address three areas:

- an all-round educated socialist personality,
- to narrow down and specify the contribution that the subculture of physical culture, sport and tourism can make,
- to develop and scientifically substantiate a system for the all-round physical training and education of socialist personalities, and provide instructions for citizens involved in leisure and recreational sports (22).

In the performance-oriented, health-oriented and controllable sports system of the SMD, leisure and recreational sports should “…be particularly geared towards the harmonization of work and leisure time of the working people of the GDR in the sense of the state leadership …” (27). There were so-called company sports communities within popular sports in the GDR. The purpose of these was to oblige companies and agricultural production companies to offer working people a diverse social, political and cultural life. The companies were therefore obliged to promote regular sporting activities for employees. In addition, the GDR launched a wide range of different measures and campaigns at the grassroots sports level, all of which aimed to encourage the population to be more active. To this end, there were popular sports campaigns and sports festivals in almost all types of sport (24).

The “Ready for Work and Defense” sports badge (“Bereit zur Arbeit und Verteidigung”; BAV complex) can be cited as a central state measure in popular sports. This sports badge was divided into a youth and an adult section. Litz (26) described the requirements for this sports badge as follows: “…In order to obtain the badge, conditions and requirements had to be fulfilled in the adult area in the sections “Social Knowledge and First Aid”, “Compulsory Exercises” (gymnastic exercise, foot march, climbing on rope or rope, swimming without time) and “Optional Exercises” (from the basic sports of swimming, gymnastics, athletics)”. The introduction of this sports badge clearly showed that those responsible in the GDR also wanted to control, influence and organize the area of popular sports. However, it was also noticeable that sport and physical activity were also a major concern for those responsible for sports at the grassroots level. Brichta's research showed very clearly that the need for defense readiness led to a transformation of the sports badge into a military sports badge, which ultimately led to the renaming of the sports badge in 1956 to “GDR sports badge—ready for work and defense of the homeland” (“Sportabzeichen der DDR-Bereit zur Arbeit und Verteidigung der Heimat”) (24) which was not always acquired on a voluntary basis (28). This non-voluntary acquisition included the compulsory acquisition of the badge in the police and army, the acquisition of the badge within school, technical school and university sports or the requirement for classification in a sport (24). With these measures, the sports management ensured that the awarding of the badge achieved supposedly high award figures, but that it was rarely worn voluntarily by the public (24). Nevertheless, popular sport played an important role in the lives of people in the GDR through the intellectual engagement with the topic of sport (social knowledge), the topic of first aid or the practice of sporting activities (compulsory exercises, elective exercises). Sport, physical culture and physical activity were omnipresent and fundamental to the health of citizens.

Another important part of popular sport was the running movement with the national “Run yourself healthy” (“Lauf Dich gesund”) campaign. This running movement was particularly popular from the end of the 1960s onwards (24), especially as the health-promoting effect of this sporting discipline was generally recognized in large sections of the population. The first state campaign of the “Run yourself healthy” campaign combined training tips, organizational matters and running events to improve the image of running (24). Hennig described the goals of this campaign as follows: on the one hand, a new target point was found and articulated with the primary position of the motif “health” and, on the other hand, a new path was taken via low-threshold offers of sporting activity, whereby the citizens were to be led to permanent action, so that the running movement as the first popular sports campaign reached qualitatively new dimensions (27). This underscored the government's efforts to offer state-sponsored popular sports and thereby promote a healthy lifestyle among the population. The “Haste by the Mile” (“Eile mit Meile”) campaign was aimed in a similar direction. This was intended to introduce sport to the everyday lives of the working population (29, 30). This instrument was aimed at the entire population with endurance activities. These included the collective completion of running and walking routes, hiking, cycling, swimming, skiing and water hiking, among others (24). However, Brichta pointed out that the ever-growing popularity of the running movement inevitably weakened due to the increasing prioritization of national high-performance sport over the years and that, as a result, other primarily exercise and sport-oriented campaigns and events were increasingly launched by the state (24). The campaigns mentioned so far were supplemented by national fitness events in a wide range of sports, such as hiking, skiing and ice skating, soccer, badminton, gymnastics, running, swimming, basketball, volleyball, table tennis, bowling and motor sports (24). These events were intended to contribute to fulfilling the state's wish of “Everyone in every place—sport once a week” (“Jedermann an jedem Ort—einmal die Woche Sport”). This slogan gave rise to a campaign of the same name, in which a total of 4.5 million participants took part (total population of the GDR in 1990: approx. 16 million), 1.3 million of whom obtained the aforementioned sports badge (20). In this context, Brichta named the annual residential area and village sports festivals, popular sports and rural sports days, sports badge days and sports afternoons at schools as further permanent state campaigns (24).

From the previous descriptions of popular sport, it is clear that those responsible for sport in the GDR also launched popular sport programs to support and promote sporting activity among the general population. This was always against the background that a healthy population was fundamental to achieving the state ideological goals of the GDR.

Remarkably, other popular sports activities based on individual initiative also developed in the GDR outside of the state-run programs. One example of these sporting events was the so-called “GutsMuths-Rennsteiglauf”. This supra-regional sporting event was virtually emblematic of the people's own sporting initiatives. The Rennsteiglauf, which was based on the mileage movement, was a long-distance and endurance run with a total distance of more than 30 kilometers. The first official Rennsteiglauf took place in 1975, which was declared a mass event with 974 participants. This event was organized without state support and financial aid. This development around the Rennsteiglauf was a thorn in the side of the state, as the state seemed to lose control over the mass sports sector, so that the state subsequently tried to prevent further sporting events of this kind from taking place (24). However, as the following years would show, the popularity, acceptance and interest in this running event grew rapidly and the event celebrated ever greater success, with over 7,000 runners taking part in 1979 (24). Due to this development, the number of participants was initially capped by the state at 7,500 and later at 9,000 people (31). Reinhart and Krüger analyzed that the fear of the state leadership and the reaction of wanting to prevent these races highlighted “…the ambivalent relationship between the programmatic claim to power of the sports leadership in leisure and recreational sports and the sporting reality …” (31) made clear and the state leadership no longer had complete, desired control over grassroots sport at this point. In response, the opportunities for amateur athletes to practice their sport were severely restricted. This mainly affected athletes who were dependent on certain materials and sports facilities. The state thus reduced the quantity and quality of the framework conditions in the sense of proportional prevention, which ultimately ran counter to health-promoting effects in the population as a whole. Very similar effects could be seen in activities such as bodybuilding, ocean sailing and martial arts.

## 4 Discussion

Through the inspection of original documents and through the topic-related primary and secondary literature, the fundamental importance of popular sport as an essential feature of the health system in the former GDR could be worked out through the establishment of the SMD (3, 5, 6, 8, 13, 18–20, 24, 25).

### 4.1 Limitations and strengths of the literature sources

Certainly, the comparatively small number of approximately 32 available literature sources limits the conclusiveness of our study, especially since access to these sometimes very old literature sources proved to be quite difficult. Nevertheless, the thematic content presented was primarily intended to provide an overview of the formal and substantive implementation of SMD in the GDR over four decades, so that less attention was paid to details in the presentation of the results obtained. Furthermore, the focus was on subject-specific content from primary and secondary sources, which largely enabled a factual and ideology-free interpretation of results.

### 4.2 The institution of SMD and popular sports

There is no doubt that the state leadership of the GDR, earlier than Western nations, recognized the importance of popular sport in terms of health policy in order to promote individual health on the one hand and to enable prevention and health promotion within the social community on the other (26). As a result, the areas of prevention and health promotion through regular and sustained physical activity represent a fundamental resource for physical and mental health (7, 10, 11, 18, 31, 32). However, within these efforts, the intention of the GDR state leadership to strengthen the state and the economy in socialist society was unmistakable (6, 7, 9, 11, 23, 26). It must also be emphasized at this point that from around the beginning of the 1970s, the SMD organization increasingly focused on ideologically oriented competitive sport at the expense of popular sport, with the corresponding allocation of resources (6, 7, 9, 22, 24, 33, 34), which increasingly weakened the primarily population-oriented prevention and promotion of health through sporting activity. The consequences for popular sport could certainly be described as blatant, as it received hardly any attention from the state leadership and severely restricted athletes in their activities.

However, this low level of state support for popular sports did not necessarily lead to less sporting activity among the population of the GDR. Opportunities and ways were found to remain active in sports on one's own initiative (28, 29). Of course, grassroots sport was also relevant for state-sponsored competitive sport (3, 7, 8, 11, 33). The special talent search and talent scouting functioned as an interface between grassroots and competitive sport, but this does not need to be discussed further at this point.

Despite many deficits and shortcomings, but also objective strengths within the institution of the SMD as part of the health care system in the GDR, it must be noted that, due to the dissolution of the SMD in 1990 (3, 9) a very profound expertise in sports medicine for the field of popular sports was lost. But, we do not have to forget the well-known health-risk effects of elite sports in the GDR—which is a clear shortcoming from a behavioral and relationship prevention perspective.

## 5 Conclusion

In critical appraisal of all topic-related documents, the complete and rigorous dissolution of the SMD regarding the health-promoting aspects of popular sport is to be classified as regrettable, especially as the health-risk potential in industrialized society tends to grow due to a rise in sedentary lifestyle. Since German reunification in 1989, there have continued to be considerable differences between eastern and western Germany in terms of promoting popular sports. The eastern German states have fallen behind the west due to sports facilities in need of renovation, i.e., a low density of sports clubs, economic weaknesses, and socioeconomic shortcomings (35, 36), which must be addressed. From a health science perspective, it is advisable to develop new initiatives for ideology-free approaches and instruments for the effective promotion of popular sport while minimizing topic-related research deficits. Against the backdrop of persistent structural disparities between eastern and western Germany more than three decades after reunification, these findings underline the need for targeted, evidence-informed policy measures to strengthen popular sport as a public health resource, particularly in structurally disadvantaged regions.

## Data Availability

The original contributions presented in the study are included in the article/Supplementary Material, further inquiries can be directed to the corresponding author.
